# American Medical Association

**Published:** 1849-07

**Authors:** 


### ARTICLE II.

### AMERICAN MEDICAL ASSOCIATION.

The late meeeting of this body, held in the city of Boston by adjournment, from the first to the fourth of May inclusive, was the largest assembly of medical practitioners ever held on this continent, if not in the world. The number of members present was four hundred and fifty four. In addition to these, a large number of the physicians of Boston and its vicinity were occasionally in attendance.

Nearly all the States of the Union were represented, from Maine, tne extreme North East, to Lousiana and Iowa in the South and West, so that it may more properly be called a National Institution than at any former period of its existance.

The arrangements made by the committee appointed for the pnrpose were admirable. The numerous public institutions of Boston and vicinity, were thrown open to the members, and the most sumptuous entertainments were given by Drs. Warren, Biglow, Hayward, Homans, and Mr. Abbott Lawrence, and by the physicians of Massachusetts at the Revere House.

Without the means of obtaining an abstract of the proceedings of the Association from the records, we can give but a synopsis from memory, and the reports published during its sittings.

We are happy to say that with the exception of a little confusion occasionally, for the want of a knowledge of parliamentary rules, (which practitioners of medicine have little use for at home) the business was transacted with the smoothness of clock-work, without the aid of *very* much labor in working the wires.

At eleven o'clock Tuesday May 1, the delegates were called to order and addressed briefly by Dr. Warren in behalf of the Massachusetts Medical Society; after which Dr. A. H. Stevens, President, delivered an address which we shall notice more fully hereafter.

A committee of one from each State represented, was appointed to nominate officers for the ensuing year, and the Association adjourned until half past three o'clock in the afternoon.

The Association met pursuant to adjournment.

Upon the nomination of the committee appointed in the morning, the following officers were unanimously elected.

President. Dr. John C. Warren of Massachusetts.

Vice Presidents. Drs. John P. Harrison of Ohio, H. H. McGuire of Virginia, Austin Flint of New York, and R. S. Stewart of Maryland.

Secretaries. Drs. Alfred Stillé of Pa., and H. J. Bowditch of Mass.

Treasurer. Dr. Isaac Hays of Pa.

A committee conducted the President to the chair, upon which he said:—

GENTLEMEN:—The honor you have now conferred on me, in electing me to be your President, I accept with unfeigned diffidence. The chair of your presiding officer has been filled by gentlemen of the highest talents and experience of whom our country can boast; and I feel myself entirely inadequate to fulfil the duties of the post you have assigned to me in a manner to do justice to the importance of this great association. On your kindness I must rely for aiding and supporting me in unravelling the difficult questions which so often arise in deliberative assemblies.

*Gentlemen*—The objects for which you are this day convened are worthy of your best attention. In the foremost rank is the improvement of medical education Education is the parent of Science, and Science it is which has led to the vast discoveries of the present day. By her aid we have seen drawn from the heavens and plucked from the deep those agents which enable us to communicate with the most distant regions, and to enjoy and exchange the products of our mutual labors. It is by education and by science that the great political revolutions of the present day are to be settled in an orderly and permanent way, so as to insure the tranquillity and happiness of the civilized world. Whence the vast difference in the conduct and consequence of the revolutions of Southern and Northern America? Is it not to be sought in the difference of education, which in the one presents us an enlightened population, and in the other the great masses in a state of ignorance, prejudice, and violence?

Medical education has sprung up in different parts of this country, according to the exigencies of different communities. There is a want of uniformity in the mode of instruction and in the qualifications for practice. It will be your business to point out, representing as you do every part of this great country, the principles on which should be founded a satisfactory medical education. If you can accomplish this, of which there is no doubt, you will apply the surest remedy to the root of the prevailing evils which spring from empiricism and falsehood.

Another object whcih will no doubt arrest your attention is the means of preventing disease. Hygiene is a department of our science which we must think has been too little studied, when we reflect how much easier it is to prevent disease than to cure it. You will perhaps consider it necessary to propose to all medical institutions that a department should be established for instruction in the means of preserving health. How important it is that the public and individuals should be better informed on this subject! In the use of articles of food for instance, which while they are so requisite to health and life, are often made the vehicles of disease!

To our profession in a good measure is to be attributed a revolution, already accomplished in part, in regard to the use of stimulant drinks, which does honor to the country and the age which produced it.

The vital air is sometimes the medium of disease. By it are transmitted the miasmata which propagate great epidemics, carrying with them desolation and death. The source of this miasmata must ever be one of the most interesting objects of research to the medical philosopher—a dark and difficult research indeed, which at first view might appear hopeless. Observation has, however, shown us one remarkable fact relating to this subject—that the great epidemics of plague, yellow fever, and cholera, have never yet invaded the regions south of the equator. What a singular anomaly in the history of natural events! Yet we find an analogy still more remarkable, because in its influence more extensive in the law which has ordained that the animal and vegetable productions of corresponding latitudes South and North of the equator scarcely ever present a similarity of species. Perhaps we may herafter find that in those Southern regions the direction of the atmospheric waves is such as to roll back from those countries the miasmata of plague, yellow fever, and cholera.

At the appearance of cholera the best efforts of our profession should be awakened. This gigantic spectre arising from the dark morasses of the East, and pursuing its course where it finds the food required for its propagation, has a second time crossed the Atlantic, and will afford us fresh opportunities of studying its character and progress, if not of discovering its origin and cure.

The cure of disease, gentlemen, is after all, your ultimate object. In the accomplishment of this, every age has flattered itself with being wiser than the past. The present may, perhaps, apply to itself, with justice, a degree of this flattery. It must, at least, have the credit of acknowledging its own ignorance, and of knowing that in order to pursue its investigations it must closely observe the course of nature in resisting the progress of disease, and in the means by which the endeavors to remove the derangements of the animal econmy. "*Natura duce*" is the motto of our Society, and guided by this maxim we are sure to arrive at results which will conduct us to a satisfatory and successful practice.

The reading of reports being in order, Dr. D. Francis

Condie, as its chairman, read part of a lengthy report from the Committee on Practical Medicine, but before it was through the reading was suspended, and the report referred to the Committee on publication. Adjourned until 10 o'clock to-morrow morning.

Wednesday Morning May 2d, 10 o'clock. The Association met pursuant to adjournment.

A motion that reading reports in full be dispensed with, and that the chairman of each committee have the privilege of reading such parts as he deemed of most interest, having passed, it cannot be expected that we should attempt even a synopsis of these valuable documents until they shall be laid before us in their printed form.

Dr. N. R. Smith of Maryland, Chairman of the Committee on Surgery, read a lengthy and able report. This document dwells at length upon the improvements in surgery, and highly approves of the use of Anæsthetic agents in general. The report was referred to the committee on publication.

After some discussion in reference to the rights of permanent members, involved in a proposed amendment to the constitution, the subject was referred to a committee. Dr. Stevens of N. Y., Wood and Condie of Phila., Arnold of Savannah Ga., and Knight of New Haven Con., were appointed.

Dr. Chandler R. Gilman Chairman of the Committee on Obstetrics, then read the report from that committee, which dwelt at length upon the use of Anæsthetics in Midwifery not only approving of their use but contending that they may not be rightfully withheld. The report was referred to the committee on publication.

The Association took a recess.

*Afternoon Session.* — The next business was reading the report of the Committee on Medical Literature, by Dr. John P. Harrison of Ohio, the Chairman.

This report attracted the attention of the association, and elicited an occasional burst of applause for its pointed hits. It was written in the peculiar style of Prof. Harrison, and although many parts of the report were omitted, enough was given to show that a large part of the field of medical science was under cultivation. A synopsis will be given hereafter. The report was referred to the committe on publication.

The report closed with some recommendations for promoting the growth of our medical literature, which were referred to a committee composed of Drs. Horner, Condie, and Hays, of Pa.

Some discussion in reference to the subject of an international copy right law, took place in this connection. And on motion of Dr. Wood, the same committee was instructed to report on that subject also. In urging this motion Dr. Wood remarked.

That it was essential to the medical literature of the country, that an international copy-right law be established. He claimed it for our own writers, who now receive no encouragement. American publishers can now procure and reprint foreign books for a less price than American authors can afford to write them. They must produce a better book, a great deal better book than the English writer, or they cannot find an American publisher who will pay them for their work. He claimed it also on the ground of justice to English writers, who were despoiled of the labour of their head and hands by the cupidity of our booksellers.

The meetings thus far had been held in the spacious Hall of the Lowell Institute, but the Hall of Representatives in the State House, the use of which had been voted to the Association, being left vacant by the Legislature, the Association adjourned to meet there at 10 o'clock to-morrow morning.

Thursday May 3d. The Association met pursuant to adjournment.

On motion of Dr. I. Hays, Dr. Agassiz was elected a permanent member.

The nominating committee, agreeably to former instructions, reported nominations for the standing committees, the chairmen of which we are able to give as follows.

On Medical Sciences, Dr. Usher Parsons of Providence R. I. Practical Medicine, Dr. J. K. Mitchell of Phila. Pa. Surgery, Dr. R. D. Mussey of Cinti. O. Obstetrics, Dr. T. G. Prioleau of Charleston S. C. Medical Education, Dr. J. Roby of Baltimore Md. Medical Literature Dr. Alfred Stillé of Philad. Pa. On Publication Dr. Hays of Philad. Pa.

A Resolution offered by Dr. A. H. Stevens of New York, creating a standing committee of seven, on each of the subjects of Forensic Medicine, Indigenous Botany and Materia Medica, and on Hygiene; was adopted

Dr. Luther V. Bell, of Boston, moved to amend the resolution so as to refer so much of the subject of Medical Jurisprudence as relates to Insanity, to a select committee. But the amendment was lost.

The committee on Forensic Medicine as appointed by the recommendation of the nominating committee, is as follows.

Drs. A. H. Stevens of N. Y. Ch. L. V. Bell of Boston. Pliny Earle of N. Y., W. F. Rockwell of Vt., R. S. Stewart of Balt., R. Watts of New York, and J. Knight of New Haven Ct.

From the difference in opinion of different members of this committee, we shall expect two reports from it, and a very thorough discussion of the subject of the Medical Jurisprudence of Insanity in crminal cases.

The other committees were appointed in like manner. Dr. Eli Ives of New Haven being chairman of that on Medical Botany, and Dr. Joseph M. Smith of N. Y., of that on Hygiene.

Dr. M. S. Taft on behalf of Dr. F. C. Stewart of New York, chairman of the Committee on Medical Education, presented and read an elaborate report, which embraced a complete account and comparison of the Medical Institutions of Europe and of this country.

It was accompanied by a series of resolutions: A paper prepared by the medical faculty of the Harvard University, giving at length reasons for not extending the lecture term beyond the usual period of four months, also accompanied the report.

Dr. Parrish offered the report of the Committee on Hygiene, for Dr. Winn chairman, who was absent. A motion to dispense with the reading was discussed but decided in the negative. Drs. Jackson of Philad., and Curtis of Mass., read papers as supplementary parts of the same report.

Adjourned to half past three o'clock P. M.

Association met pursuant to adjournment, and resolved itself into committee of the whole for the consideration of the resolutions recommended by the Committee on Medical Education. Dr. Knight of Ct. in the chair. The resolutions were considered seriatim as follows.

1st. *Resolved*, That the attention of Medical Colleges be again directed to the resolutions of the Committee on Preliminary Education adopted by the Medical Convention of 1847, and that they be advised to require from students that they shall in all cases produce certificates of preliminary education. *Carried*.

2d. *Resolved*, That the several State and County Societies, as well as all other voluntary Medical Associations throughout the country, be advised and requested to adopt the plan proposed by the Medical Society of the State of N. York, at its last annual meeting, for ensuring due attention to the subject of preliminary education.

Dr. N. S. Davis, of New York, explained that the plan of the New York State Society was, that every County Society should appoint a board for preliminary examinations of students, with a view that they should be required to produce certificates from such boards before they could be received as medical students in the office of any private practitioner.

Dr. Evans, of Chicago Illinois, offered the following as a substitute.

*Resolved*, That as students are generally introduced to the profession by private preceptors, it is recommended that no students be received by them unless they come up to the standard of preliminary education precribed by this Association.

Dr. Evans remarked that he had no objection to the plan of the New York State Medical Society except its complication. It no doubt will work well and do good in New York where the profession is completely organized, but in a large number of the States no such organization exists.

The substitute offered strikes at the root of the evil about which we have heard so much complaint against the Medical College. If we want the standard of attainments in the profession elevated, we must select the materials of which we make physicians. The only way to do this is to guard the portals of the profession—not to place the sentinel at the door of the sanctum. Rigid examinations in the green-room will not answer, at least in the West. After a student has spent the time of his pupilage in his preceptor's office, he considers himself a doctor, and the question is not mooted whether he shall go to practice or not, but whether he shall attend lectures previously or go to practice without; and if the Colleges will not admit him for want of preliminary education, the result is he sets up on his own account.

Dr. Bond of Baltimore followed in favor of the amendment. He went into a lengthy discussion of the standing of the profession of Medicine with the people generally. He thought we made quite a mistake in attaching so much importance to the colleges. The standing of the profession was such that a certificate of qualification from a fashionable clergyman would do more towards introducing one to practice than any college diploma. Thus the miserable quack was often introduced to the parlor, and made the object of the most polite attention, while the worthy yet modest man of of science was doomed to tread his thorny path through the shades of neglect.

Dr. Warren, of Boston, thought the picture of the profession by the last gentleman over-wrought. He thought the profession stood high, and that it was the best profession in existence, that ever did exist, or ever would exist. After remarks by others that were not gathered, the amendment was adopted by a large vote.

3d. *Resolved*, That this Association does not sanction or recognize "College clinics" as *substitutes* for Hospital clinical instruction, and that the Medical Colleges be again advised to insist in all instances where it is practicable on the regular attendance of their pupils during a period of at least six months upon the treatment of patients in a properly conducted Hospital or other suitable institution devoted to the reception and cure of the sick.

The resolution was adopted.

4. *Resolved*, That it would conduce both to the convenience and advantage of the students, if the subjects taught in the Colleges were divided into two series; the one of which should be studied during the first year's attendance on lectures; and the other, during the second session. And that examinations should be instituted at the close of the first course of lectures on the subjects taught during that course, certificates of which should be required prior to the final examination. Rejected.

5. *Resolved*, That it is the deliberate opinion of this Association that the plan of examining students for medical degrees in private, and before one professor only at a time, is highly defective, and should be at once discontinued. Laid upon the table.

6. *Resolved*, That examinations for medical degrees should be practical, and that it is desirable as far as practicable that they should be conducted in writing as well as *viva voce*. Laid on the table.

7. *Resolved*, That in view of the importance of a due knowledge of practical pharmacy, the medical schools be advised to require from candidates for degrees that they should produce satisfactory evidence of their having been engaged in compounding medicines and putting up prescriptions, either under the direction of their private preceptors, or in the shop of a recognized and qualified apothecary. Laid on the table

In regard to examining boards and licenses:

8. *Resolved*, That the interest both of the public and the medical profession would be promoted by the establishment of boards of examiners in each of the States of the Union, to examine candidates for licenses to engage in the active practice of medicine and surgery. Laid upon the table

9. *Resolved*, That the examiners should satisfy themselves that condidates are familiar with the elementary branches of general knowledge. Laid on the table.

10. *Resolved*, That for the purpose of carrying out the objects contemplated in the foregoing resolutions, a special committee of seven members be appointed to prepare a memorial and form of law in reference to the subject of the establishment of boards of medical examiners to be submitted to the Association at its next annual meeting. Indefinitely postponed.

The Committee rose and reported, when the whole subject was, on motion of Dr. Stevens, committed to a special committee to report to-morrow morning. The Association adjourned.

Friday Morning. The Report of the Special Committee on Indiginous Medical Botany being called for, Dr. N. S. Davis of N. Y., Chairman of the Committee, stated, that a report had been prepared by himself in continuation of the one published in the transactions of the society last year. He stated that there were no less than 1000 plants in our country, reputed to posess medicinal properties of value in the treatment of disease; but out of this whole number, only about 150 have been so investigated as to rank among the well known articles of the Materia Medica. And of the large number which remains, our knowledge is of the most vague and indefinite character; thus out of about 280 indiginous and naturalized plants named in Griffith's late and valuable work on Medical Botany, concerning 150, we are merely told that they are *said* to be good in this disease or that, or they are *said* to have been used by the *Indians*. He further stated that in making up his report, he had chosen such a number of articles, only, as could be thoroughly investigated in reference to the following questions, viz., 1st, On what part of the system does the remedy make its impression?

2d, What is the nature of such impression, and what its secondary effects? 3d, In what ingredients of the plant does its active qualities reside? The committee had acted on the principle, that it was better to settle the real value and modus operandi of one article only during the year, than to collect an unreliable evidence concerning a much larger number.

The report was composed in part of two other papers, prepared with great care and labor by S. W. Williams, of Deerfield Mass., and Dr. F. P. Porcher of Charleston S. C., both members of the committee. Their papers contained a complete catalogue of the medicinal plants of their respective states, arranged according to their natural order. the whole report was referred, without reading, to the Publishing Committee.

Dr. Stevens chairman, reported from the Select Committee appointed last evening, the following resolutions.

1st. *Resolved*, That the Association reiterate their approval of the resolutions in reference to medical education, adopted by the Convention, which met in Philadelphia, in May, 1847, and contained in pages 73 and 74 of the published proceedings of that Convention.

2d. *Resolved*, That the attention of Medical Colleges be again directed to the Resolutions of the Committee on Preliminary Education, adopted by the Med. Convention of 1847, and that they be advised to require from their students that they shall, in all instances, present certificates of due preliminary acquirements prior to graduation.

3d. *Resolved*, That physicians throughout the Union, be advised and requested to require of those wishing to become their pupils, evidence of a proper general education, before admission into their office.

4. *Resolved*, That the Association does not sanction or recognize "College Clinics" as substitutes for Hospital clinical instruction, and that the Medical Colleges be again advised to insist, on the regular attendance of their pupils, during a period of six months, upon the treatment of patients in a properly conducted hospital, or other suitable institution, devoted to the reception and cure of the sick.

5. *Resolved*, That in accordance with a resolution of the American Medical Association, adopted May 4th, 1847, "it as earnestly recommended to the physicians of those States in which State Medical Societies do not exist, that they take measures to organize them before the next meeting of this Association.

6. *Resolved*, That the State Societies be recommended, after they shall have been organized, to recognize as regular practitioners none who have not obtained a degree in medicine, or a license from some regular medical body.

7. *Resolved*, That the Association recommend to the various Schools of Medicine to meet at Cincinnati before the next annual meeting of this Association, and present a plan for elevating the standard of medical education, to this Association.

The chairman hoped that they would be adopted without division.

Dr. Harrison moved that the report and resolutions be adopted.

The motion was opposed warmly. Dr. N. S. Davs, isaid we sit day after day listening to the reports of committees, have subjects recommitted and passed upon without any consideration by this body, and when the great subject of medical education is taken up for consideration, it is proposed to restrict members in their remarks.

On motion of Dr. R. D. Arnold, of Georgia, the Association resolved itself into committee of the whole, to consider the resolutions seriatim. Dr. Arnold in the chair.

The ten minutes rule was adopted.

The first, second, and third resolutions were adopted.

The fourth resolution caused much discussion, but a small part of which we are able to report.

Dr. Jackson argued at length the impropriety of governors of hospitals excluding students from the wards of such institutions. He said that in Philadelphia they had to adopt the College clinics, as they had for a few years past been excluded from the great City Hospital on the most trivial ground, the mere cracking of a cock-roach being the foundation of the whole affair. This left them as the best alternative, a resort to ambulatory patients.

Drs. Pattison, Harrison, Gibbons, Davis, and others participated in the discussion of the resolutions. It was proposed to amend the fourth resolution so as to read, "while the Association admit the value of College clinics &c.," but the motion was lost.

After amending the resolutions reported by adding, on motion of Dr. Thos. E. Bond Jr., the following, they were reported back to the Association, and adopted.

*Resolved*, That this Association recommend the encouragement of private medical institutions, strongly advising that dispensary practice be made, as far as practicable, a part of the means of instruction.

Dr. Stillé offered a highly complimentary resolution expressive of the lively sense of gratification felt by the Association for the many marks of kindness it had received during the session. Adopted.

Dr. Bowditch offered several resolutions of thanks, which were adopted.

Dr. Hays, from the committee appointed to consider the recommendations of the Committee on Medical Literature, reported in part the following.

*Resolved*, That a committee of three be appointed to memorialize Congress in favor of an international copy-right law. Amended so as to require the committee to report to the next meeting. Adopted. The President appointed Drs. G. B. Wood, T. E. Bond, Jr., and I. Hays.

Dr. Evans of Chicago, offered the following Preamble and Resolution, which were adopted by a very large vote.

Whereas, Merit should be the test by which one individual is preferred to another, *And Whereas*, the places of profit and honor in our profession should be open to the competition of all, in order that the best selections may be made, therefore,

*Resolved*, That trustees and others, exercising the office of appointing Professors in Medical Schools, be requested to adopt the system of *Concours*, or public trials, among the means resorted to for calling out the talent of the profession and ascertaining the qualifications of applicants.

Dr. G. B. Wood offered a preamble and resolution in reference to the paper of the faculty of Harvard University on the length of College sessions. But before action was taken the Association adjourned until half past three o'clock.

Afternoon Session. The resolution offered by Dr. Wood in the morning was adopted, in pursuance of the provisions of which, Drs. Jackson, J. L. Atlee, and Alfred Stillé, of Penn. were appointed a committee to prepare a paper in favour of lengthening the Sessions of College Lectures, to be printed with the one above referred to.

On motion of Dr. Hays the several standing Committees were instructed to stick to the text. Dr. H. also offered an amendment to the Constitution, abolishing the standing Committees, which by rule lies over until next year.

Dr. Ware, of Boston. offered a resolution instructing one of the standing committees to enquire into the expediency of using the English language exclusively in writing prescriptions &c., which was adopted.

Dr. Wood gave an account of his reception as delegate to the British and to the Provincial Medical Associations, and brought the compliments of the latter body, by their request.

On motion of Dr. Johnson, of Mo., the Committee on Education were instructed to enquire into the expediency of establishing Schools of pharmacy, and adopting a rule that physicians do not patronize druggists who deal in nostrums.

After various resolutions of thanks to the officers and others who had served the Association, and congratulatory speeches from Dr. Z. B. Adams, of Boston, and others, the Association adjourned *sine die*, the place and time of the next meeting having been previously fixed on the first Tuesday in

May next at Cincinnati. Dr. Drake and others of that place, were appointed a Committee of Arrangements.

Thus closed, in harmony, a meeting, which, for talent and erudition, has seldom been equaled. Its influence must be powerful in forming the character and opinions, and in controling the practices of our profession. E.

